# Room for Improvement in Sports Nutrition Knowledge amongst Parents and Caregivers of Male Academy Soccer Players in the UK: A Cross-Sectional Study

**DOI:** 10.3390/nu15204331

**Published:** 2023-10-11

**Authors:** Luke Callis, Mark Russell, Howard Hurst, Jack Hardwicke, Charlie Jon Roberts

**Affiliations:** 1 Faculty of Arts, Science and Technology, University of Northampton, Northampton NN1 5PH, UK; luke.callis@northampton.ac.uk; 2 School of Sport and Wellbeing, Leeds Trinity University, Leeds LS18 5HD, UK; m.russell@leedstrinity.ac.uk; 3 Centre for Applied Sport Physical Activity and Performance, University of Central Lancashire, Preston PR1 2HE, UK; hthurst@uclan.ac.uk; 4 Department of Sport Science, Nottingham Trent University, Nottingham NG11 8NS, UK; jack.hardwicke@ntu.ac.uk

**Keywords:** nutrition knowledge, academy soccer, nutrition support, sports nutritionist, youth nutrition, youth athlete

## Abstract

In professional soccer clubs in the UK, nutrition support is often polarised towards senior players or those in the senior academy age groups seeking first team selection/involvement. Accordingly, there is an increased reliance on parents and caregivers to provide support regarding nutritional intake. Therefore, the aim of this study was to evaluate the quality of nutrition knowledge of parents and caregivers of male youth soccer players within developmental academies in the UK. Across a single competitive season, 360 registered parents and caregivers of male soccer players from English Premier League under 9–11 (Foundation Phase) and under 12–16 (Youth Development Phase) age group academies completed an online version of the 88-item validated Nutrition for Sport Knowledge Questionnaire. Nutrition knowledge scores were classified as “poor” (43 ± 23%). Nutrition knowledge scores were significantly greater in respondents with dependents representing Category 1 (45 ± 13%) vs. Category 2 (39 ± 15%) academies and in Foundation Phase (44 ± 13%) vs. Youth Development Phase (41 ± 15%). These results demonstrate that there is room for improvement in the nutrition knowledge levels of parents and caregivers of male soccer academy players. We recommend that club academies provide appropriate resources towards nutritional education for parents and caregivers.

## 1. Introduction

The Elite Player Performance Plan (EPPP) is a strategy introduced in 2012 by the English Premier League to develop high-quality homegrown male soccer players by increasing investment in youth development [1]. Age ranges of youth academy players in the EPPP Foundation Phase (FP), Youth Development Phase (YDP), and Player Development Phase (PDP) are under (U) 9–11 years, U12–16 years, and U17–23 years, respectively. In the 21/22 season, there were 14,226 players enrolled in the EPPP system, with FP and YDP players totalling 3741 and 7226, respectively [2]. Clubs are categorized from Categories 1 to 4, with Category 1 clubs providing more comprehensive services and receiving the most funding [1].

Soccer players require careful manipulation of their overall energy, macronutrient, and micronutrient intakes, along with consideration for daily and weekly variability in food intake in relation to training and matches [3]. English academy players exhibit large but variable energy expenditures due to the high-intensity, intermittent nature of play [4], high weekly distances covered during training and matches [5], and total activity duration [4,5]. The number of training sessions and match events players engage in weekly increases from four in Under-9s to nine in under-16s age groups, with some days containing ≥ two sessions [4]. Using the doubly labelled water method, total energy expenditure of Category 1 academy players was measured over a 14-day in-season period [5]. The authors observed energy expenditures of 2859 ± 265 kcal·d^−1^ and 3029 ± 262 kcal·d^−1^ in the U12/13 and U15 age groups, respectively. High daily energy expenditures may increase susceptibility to negative energy balance unless matched with appropriate nutritional intake, with mean daily deficits of −311 ± 397 kJ reported in U16 male Premier League academy players [6]. Furthermore, youth academy players will likely have attendance at school, social obligations, and rest periods as priorities alongside training and matches [7]. Therefore, youth academy soccer players not only have high-energy demand due to sporting commitments but also have congested schedules that can lead to difficulty in meeting energy requirements; thus, nutritional support from clubs and/or stakeholders is important in this context.

There is variability in the type and depth of nutrition services required by clubs. Notably, the EPPP stipulates that Category 1 clubs are required to employ a SENr accredited nutritionist or be under the management of an individual with this accreditation on at least a part-time basis, with Category 2–4 clubs not having any requirement for nutrition support personnel [8]. Full-time nutritionists are provided by 64%, 0%, and 14% of Category 1, Category 2, and Category 3 academies, respectively [9]. The types of nutrition support offered include individual consultations and group seminars, support for stakeholders including support staff and caregivers, and cooking sessions across Category 1–3 academies [9]. Some clubs provide main meals, snacks, and beverages to all players, some offer meals sporadically, some providing meals to older players enrolled in the PDP to a much greater extent than younger players, whilst some provide no meals at all [9]. Collectively, it is clear there is a skewed approach to nutrition support provision, with efforts focused on higher age groups overall but with more concentrated effort in Category 1 academies.

Sports nutritionists indicate that limited time is often a barrier to providing adequate support, particularly given that full-time roles are rare and that the number of hours practitioners are employed does not translate to contact time with players and key stakeholders [10]. As the dietary habits of children in the FP and YDP age groups are heavily influenced by the parents’ and caregivers’ (“caregivers” hereafter) purchasing, provision, and preparation of food and meals [11,12,13] on an almost full-time, exclusive basis, such stakeholders are key influencers of the quality of food provided.

Dietary habits are multifactorial and are influenced by socioeconomic status, education, cultural background, appetite, cost, convenience, and knowledge [14,15]. Nutrition knowledge is described as an important factor in the food choice process of athletes as this can influence not only an awareness of nutrition but also the ability to apply this practically when choosing appropriate food and meals [16]. Those possessing greater levels of knowledge tend to consume more fruit and vegetables [17,18], and athletes with higher nutrition knowledge scores demonstrate more favourable body composition values [19]. Whilst described previously in elite youth mixed athletes [20,21], the nutrition knowledge levels of the caregivers are likely of major importance. Lack of nutrition knowledge in caregivers was identified as a barrier for adolescents receiving healthy meals [22], and adolescents described family members as important sources of information on nutrition, with their support also one of the most helpful factors for promoting dietary change [23]. Furthermore, older academy players highlighted that caregivers can be enablers to good dietary habits if their nutrition knowledge is good; however, the requirement to eat what is provided for the family may be a barrier to players-specific nutrition requirements being met [24]. Despite the important role caregivers play in helping children and adolescents adopt appropriate nutritional practices, a recent audit into nutrition services provided by English soccer academies indicates that although 50–90% of clubs in Categories 1–3 reported providing “parent education”, collective FP and YDP nutrition support totalled <20 h per month [9].

It was previously highlighted that clubs should allow adequate time for sports nutritionists, ideally on a full-time basis, to provide sufficient support for all age groups in academies beginning at a young age [24]. With a part-time nutritionist being required only when academies possess Category 1 status, ensuring adequate nutrition support is provided to all players enrolled from U9–U23 and key stakeholders may be unfeasible. Given the importance of nutrition for health and well-being and athletic success and development in youth players and the critical role caregivers play in supporting this, it is important to understand whether these individuals are best equipped to facilitate optimal dietary practices. As such, the purpose of the present study was to quantify the nutrition knowledge of the caregivers of elite FP and YDP male academy soccer players, compare overall scores from Categories 1 to 3 academies and age-stratified phases, and report overall question-specific responses relevant to the population. We hypothesised that caregivers would present “poor” nutrition knowledge levels due to the limited nutrition support offered to caregivers in academy environments. 

## 2. Materials and Methods

### 2.1. Study Design

A total of 364 individuals who were registered as parents or primary caregivers to male players within the FP (U9–U11) or YDP (U12–U16) were invited to participate in this study. Clubs involved were of Category 1, Category 2, and Category 3 status (as per 22/23 season classifications). Data collection was conducted between November 2022 and April 2023. Ethical approval was obtained from the University of Northampton Faculty of Arts, Science and Technology Research Ethics Committee (FAST-REC approval number: 212211).

This study utilized a cross-sectional design. Member(s) of support staff at clubs were contacted to explain the purpose and requirements of this study. If interested, the member(s) of staff were asked to distribute the questionnaire, which was accessible via a link or QR code, to caregivers of players enrolled in the EPPP academy. Prior to the questionnaire, participants were presented with the participant information sheet and consent form, with contact details of the research team if they wished to ask any questions about this study. If participants selected that they did not provide informed consent, they were taken out of the questionnaire and were not asked to provide any further information.

### 2.2. Instruments

General and sport-specific nutrition knowledge levels were assessed using the Sport Nutrition Knowledge Questionnaire (SNKQ) [25]. Demographic and general subject-specific information was enquired about prior to the SNKQ. The SNKQ comprises 88 questions covering a range of topics related to weight management, macronutrients and micronutrients, sport- and athletic-specific information, supplements, and alcohol. A correct answer received one point whilst an incorrect or “not sure” response received zero points. Overall scoring was assessed as described previously [26], being “poor” knowledge (0–49%), “average” knowledge (50–65%), “good” knowledge (66–75%), and “excellent” knowledge (75–100%). The questionnaire demonstrated both construct and content validity, and all sections except for the “supplementation” section demonstrating acceptable test–retest reliability [25]. Small modifications were made to some questions to ensure relevance to a UK population. For example, questions related to alcohol consumption were amended to reflect the UK government recommendations regarding alcohol units [27]. The item response formats include agree/disagree/not sure, multiple choice, and effective/not effective/not sure. The questionnaire was inputted and completed on an online survey-hosting website (Microsoft Forms, Microsoft, Redmond, WA, USA).

### 2.3. Statistical Analysis

Descriptive statistics were presented as mean ± standard deviation. Data were analysed using SPSS Statistics (version 28.0.1.1, IBM, Armonk, NY, USA). Shapiro–Wilks testing was applied to test for normality, with data deemed parametric. Homogeneity of variance was assessed using a Levene test. Differences in nutrition knowledge scores between academy categories were analysed using a one-way analysis of variance, with post hoc testing conducted using a Tukey HSD and *n*^2^ effect sizes interpreted as small (*n*^2^ = 0.010), medium (*n*^2^ = 0.060), and large (*n*^2^ = 0.140) [28]. An independent samples t-test was applied to explore differences in nutrition knowledge scores between age-stratified phases, with Cohen’s d effect sizes interpreted as small (*d* = 0.2), medium (*d* = 0.5), and large (*d* = 0.8) [28]. Confidence was set at *p* < 0.05 for all tests.

## 3. Results

The questionnaire was accessed by 364 individuals. Of these, three did not provide informed consent and one participant did not provide adequate information related to the academy that they were registered with and were subsequently removed from the analysis. As such, 360 participants from eleven academies (three Category 1, three Category 2, and five Category 3) completed the full questionnaire. The questionnaire was completed in an average of 27 min 36 s. Participant demographics are displayed in Table 1.

**Figure 1:**
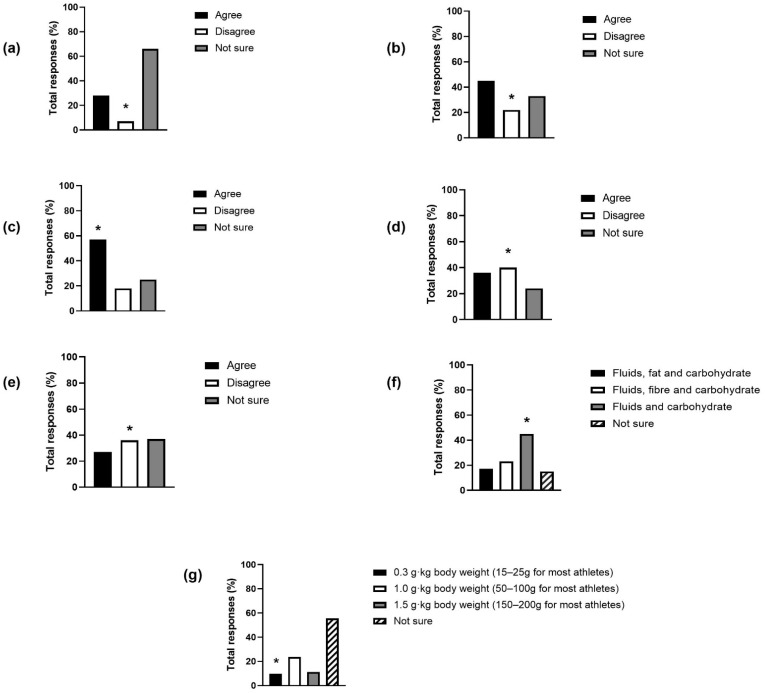
Responses (%) to questions and statements of population relevance from the SNKQ. * denotes correct response. (**a**) Athletes aged 15 to 24 years need 500 mg of calcium each day; (**b**) Iron tablets should be taken by all athletes who feel tired and are pale; (**c**) Supplement labels may sometimes say things that are not true; (**d**) All supplements are tested to make sure they are safe and don’t have any contamination; (**e**) Athletes should not eat more than 20 g of fat per day; (**f**) Before competition, athletes should eat foods that are high in; (**g**) How much protein do you think experts say athletes should eat after resistance exercise?

Collectively, nutrition knowledge scores were 42.8 ± 23.4%, classified as “poor” [26]. Thirty-six (10%) respondents indicated that they had previously received formal human nutrition education. Participants indicated that “family” (*n* = 162), “coach” (*n* = 125), and “friends” (*n* = 80) had previously given their child/dependent advice regarding their diet. The top sources of information participants relied on regarding nutrition were selected as “internet search” (*n* = 211), “family” (*n* = 183), and “coach” (*n* = 103). The organisation their child/dependent is with providing no access to nutrition support for either themselves or their child/dependent was reported by 126 participants. Collective responses for population-specific questions are displayed in Figure 1.

**Figure 2:**
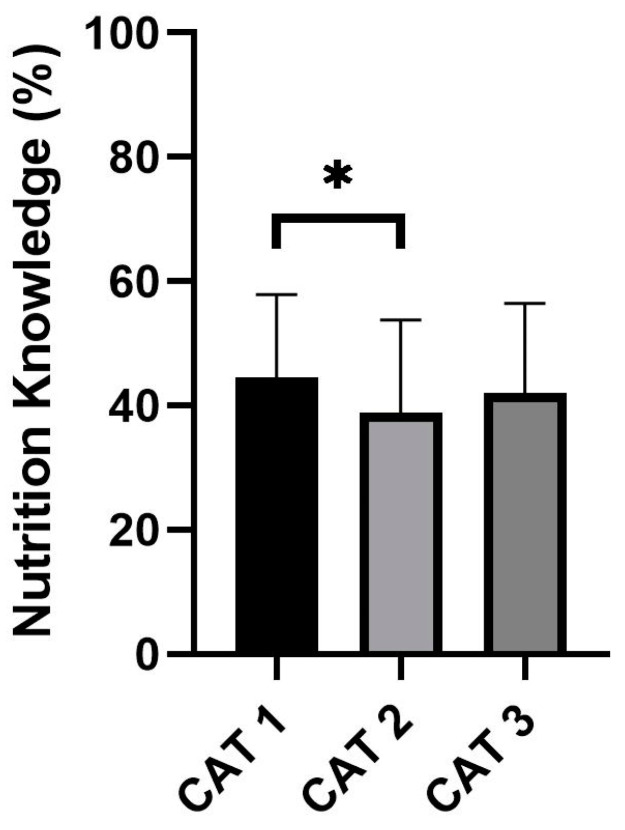
SNKQ scores between academy categories. * denotes significant difference between conditions (*p* < 0.05). CAT: category.

One-way ANOVA testing revealed a significant difference in nutrition knowledge scores between academy categories (*F* = 3.018; *p* = 0.05; *n*^2^ = 0.017; Figure 2). Post hoc analysis indicated that the caregivers of players enrolled in Category 1 academies demonstrated significantly greater nutrition knowledge scores than those enrolled in Category 2 academies (mean difference = 5.76%; *p* = 0.039). No significant differences were observed between Category 1 and Category 3 (mean difference = 2.66%; *p* = 0.355) and Category 2 and Category 3 academies (mean difference = 3.10%; *p* = 0.246).

**Figure 3:**
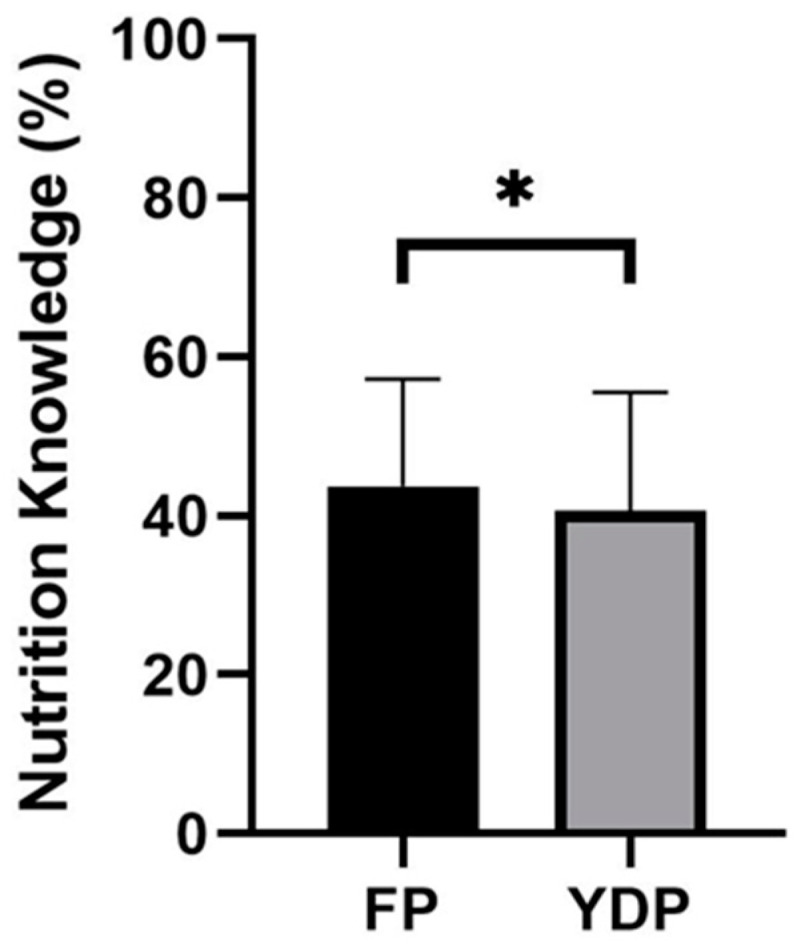
SNKQ scores between age-stratified phase. * denotes significant difference between conditions (*p* < 0.05). FP: Foundation Phase (Under 9–Under 11); YDP: Youth Development Phase (Under 12–Under 16).

Independent samples *t*-testing revealed a significant difference in nutrition knowledge scores between caregivers of players representing YDP and FP academies *(t* = 2.015; *p* = 0.045; *d* = 0.217; Figure 3). 

## 4. Discussion

The aims of the present study were to quantify and compare nutrition knowledge of the caregivers of EPPP male academy players, compare overall scores from Categories 1 to 3 academies and age-stratified phases, and report overall question-specific responses relevant to the population. In line with our hypothesis, collective responses indicate a “poor” level of nutrition knowledge in this population. Nutrition knowledge scores were greater in caregivers of Category 1 compared to Category 2 players, while responses between Category 1 and Category 3 and Category 2 and Category 3 were not statistically different. Furthermore, caregivers of FP players demonstrated significantly greater nutrition knowledge scores than caregivers of YDP players.

Overall, category-specific and age phase-specific nutrition knowledge scores were classified as “poor” [26]. Similar results using the validated 88-item NSKQ were observed in female collegiate volleyball [29], female [30] and male Australian rules football [26], and elite and nonelite Gaelic football [31] players. Professional soccer players from a variety of countries previously demonstrated an average nutrition knowledge score of 52% [32]. Despite numerous studies exploring nutrition knowledge, the present study is the first to explore the nutrition knowledge levels of caregivers of academy players aged U16, who act as key stakeholders in the delivery of nutrition practices and development of nutrition behaviours for young players.

These results are not unexpected, given the limited collective support provided within YDP and FP academies when compared to within PDP programs [9]. In the present study, 35% of respondents reported that they or their child/dependent received no nutritional support from the club. It was indicated that overall, monthly support provided from an accredited nutritionist was reported as 62 ± 57 h for Category 1 academies, 12 ± 9 h for Category 2 academies, and 14 ± 26 h for Category 3 academies [9]. Within this, “parent education” was provided within FP (Category 1: 85%; Category 2: 67%; Category 3: 56%) and YDP (Category 1: 85%; Category 2: 72%; Category 3: 59%) clubs in a skewed fashion. At the FP and YDP levels, 20 h per month of nutrition support was reported from accredited nutritionists in Category 1 academies, < 10 h per month in Category 2 academies, and <15 h per month in Category 3 academies. The support available for PDP players was reportedly greater than that for players in U9–U16 age categories both overall and with consideration to specific services such as one-to-one support and cooking workshops [9]. Whilst expected due to players in the PDP being supported to transition into senior teams, the limited support available to younger players may compromise their biological and sport-specific development and therefore their progression through adolescence towards professional contracts [33].

Ensuring energy and nutrient intake is sufficient to support optimal health, growth, development, performance, and recovery is of the utmost importance; however, academy players are likely to face challenges in meeting elevated energy requirements. Congested schedules due to sporting, academic, and life commitments resulting in limited time; as well, financial barriers were identified as challenges for athletes’ adequately fuelling by both players and coaches [34]. Furthermore, knowledge regarding appropriate dietary practices for high-level players was reported to be limited [34]. It was demonstrated that a moderate positive correlation between sport nutrition knowledge, energy (*rho* = 0.31, *p* = 0.04), and carbohydrate intake (*rho* = 0.32, *p* = 0.04) exists in professional and semiprofessional male soccer players in Australia [35]. Whilst the current study assessed the nutrition knowledge levels of caregivers, as key stakeholders in the provision of food and beverages to youth players, establishing a relationship between nutrition knowledge levels in this population and adequate dietary intake to support optimal health and well-being, performance, and recovery warrants further investigation.

Daily macronutrient requirements are elevated in youth players to provide energy and substrates for growth, development, and activity. Whilst protein and carbohydrate intake receives considerable attention in players, dietary fat is necessary for meeting essential fatty acid and fat-soluble vitamin requirements, providing energy during growth [36] and due to the greater fat oxidation rates exhibited in youth compared to adults [37]. In the present study, the statement “athletes should not eat more than 20 g of fat per day” was incorrectly answered by 64% of respondents. The UK government recommend that males aged 11–18 consume 35% of total energy intake as dietary fat, equating to an average of up to 97 g per day [38]. Additionally important is the periodization of food intake prior to matches to ensure adequate glycogen stores and hydration status [39,40,41] and following matches and training to facilitate optimal glycogen repletion, muscle damage repair, and adaptation [41,42]. Consuming foods that are high in fluids and carbohydrates before competition and protein recommendations of 0.3 g·kg^−1^ body weight after resistance exercise were correctly selected by 45% and 10% respondents, respectively. Whilst these results in isolation cannot be used to suggest that academy soccer players are observing suboptimal fuelling and recovery practices, they may highlight areas for nutrition practitioners to consider when working with players and key stakeholders such as caregivers.

Calcium requirements are elevated during adolescence to compensate for the greater bone remodelling that occurs during this life stage, with 26% of adult total bone mineral accretion expected in the two years around peak bone mineral velocity age of 14.1 ± 0.95 years in males [43]. As such, it is recommended that adolescent athletes consume 1000–1300 mg calcium daily [3,44,45]. In the present study, 7% of respondents were able to correctly identify that “athletes aged 15 to 24 years of age require 500 mg calcium per day” is incorrect. Soccer players are at an increased risk of injury due to the high-intensity, intermittent style of play and potential impacts encountered during match-play. Higher incidence of injury has been reported in youth soccer players between the ages of 13.5 and 14.5 years old, in line with athletes entering a period of rapid growth known as peak height velocity [46]. This window of increased injury risk further highlights the need to optimise nutritional strategies, particularly in the YDP age groups. Low calcium intake has been identified as a predictor for low bone mineral density in male adolescent athletes and demonstrates a cumulative effect of low bone mineral density risk when present with other risk factors such as stress fracture history and <85% expected body mass [47]. Low bone mineral density is a risk factor for fractures [48], and fractures represent 3–7% of injury occurrence within English academy environments [49,50]. Despite the low incidence compared to other injuries, fracture recovery required a median of 32 days recovery before return to play is observed in professional soccer players [51]. As such, ensuring nutritional practices are appropriate to support healthy growth and development and reduce the risk of injury is of importance for key stakeholders of academy soccer players.

The use of dietary supplements is commonplace in athletic populations [52]; however, sporting organisations including the International Olympics Committee [45] and Sports Dieticians Australia [44] discourage the use of ergogenic supplementation in adolescent athletes due to limited data, with UK Anti-Doping stating that “there are no guarantees that any supplement product is free from banned substances” [53] with 38% of 66 sports supplements sold online containing undeclared doping substances [54]. Contamination of sports supplements is unlikely to be intentional [55]; however, UK Anti-Doping rules stipulate that it is the athletes’ responsibility if they receive a positive test for a prohibited substance and recommend only purchasing batch-tested supplements to mitigate the risk of ingesting a contaminated product [53]. In the present study, 60% of caregivers did not correctly identify that not all supplements are tested to make sure they are safe and do not have any contamination. It is crucial that caregivers are aware of their responsibilities and those of their child or dependent, given that youth players registered with the Football Association in the UK are subject to strict antidoping testing regulations [56].

### 4.1. Strengths and Limitations

To our knowledge, this is the first time the validated NSKQ was distributed to caregivers of youth academy athletes; however, it is noteworthy that the questionnaire is not validated within an English population [25]. Despite its novelty and contribution to knowledge, there are limitations to this work which we acknowledge. First, we were unable to collect data on the number of registered caregivers at clubs. As such, we are not able to determine the overall response rate from those whom the questionnaire was initially presented to. Second, due to the cross-sectional nature of this study, our results only represent a specific cohort of caregivers during one competitive season. Third, the self-report nature of the questionnaire inherently leaves a possible margin of error in participant responses due to misunderstandings of the questions and social desirability bias. Fourth, despite the large sample size and recruitment of participants from 11 academies, there is the potential for selection bias due to the sampling strategy adopted. Finally, the quantitative nature of the data also means that we provide a cursory but not a rich set of data on caregivers’ nutrition knowledge. Human knowledge, and subsequent behaviour based on that knowledge, is a complex social process that cannot be effectively understood through quantitative measures alone. Future research is required to understand more fully the nutrition knowledge and behaviours of caregivers in soccer environments.

### 4.2. Practical Considerations 

Whilst data collected via the SNKQ suggests poor nutritional knowledge in caregivers, we cannot unequivocally conclude a direct translation into a lack of best-practice sports nutrition information by caregivers of youth soccer players or an inability to apply this when providing food, beverages, and meals to their dependent. Additionally, the lack of uniform responses means the practical relevance of significant differences in service provision between age groups or categories is unlikely. Despite this, our results do provide a basic insight that practitioners can consider within their practice. For example, ensuring that caregivers of YDP and FP players are provided with information about the dietary requirements of calcium for an adolescent player, alongside ways this can be incorporated into daily dietary intake, may contribute to optimal growth and development, and reduce the risk of growth and bone-related injuries. Given the negative correlation between injury to young players and progression into a first team environment [57], any strategies with potential to reduce injury risk in elite youth soccer deserve great consideration.

During childhood and adolescence, humans experience significant physiological, metabolic, and hormonal changes, with dietary intake playing a major role in the biological maturation of physiological systems that can influence health in later life [58]. Nutrition support in elite environments is often about improving performance and providing food, not supporting behaviour change or promoting optimal long-term health and well-being [10]. Relationships and behaviours with food are developed during childhood and adolescence [36], and ensuring that players and key stakeholders are provided with holistic nutrition support can help promote life skills; this is noteworthy, as the Youth Development Rules indicate that Category 1 and Category 2 academies in the EPPP are required to provide, independent of a registered nutritionist, individuals on a full-time and part-time basis, respectively, to develop life skills such as those surrounding food and nutrition [2]. This is important to consider not only due to the immediate impact that suboptimal dietary intake can have on growth and development during childhood and adolescence but also given how few players will proceed to first teams [59], with 10% progression rates being reported from academy systems to senior teams [33,60]. Providing individualised support to caregivers and working within their sociocultural environment is crucial for facilitating appropriate nutritional practices in children and dependents [15]. Providing support to reenforce or improve the health and nutrition literacy of caregivers can thus extend to their dependents, promoting health behaviours over the lifespan [61].

Parents, caregivers, and wider family networks have been identified as both barriers and enablers to optimising eating patterns in academy soccer [24] and high-level rugby players [13,62]. As such, we recommend that club academies provide appropriate resources towards nutritional education for caregivers, within the means available.

## 5. Conclusions

In conclusion, these results suggest that there is room for improvement in the nutrition knowledge in the caregivers of EPPP FP and YDP academy players. Our findings highlight the importance of providing holistic nutritional support to both youth academy players and key stakeholders, as the gaps in knowledge are not only sport-specific but also pertain to nutrition for general health and growth. Despite the focus of this study on soccer, these findings have implications across any developmental athletic environments. Future research should aim to build upon these findings, using a range of methodological approaches, such as interventions designed to upskill caregiver nutrition knowledge and qualitative data collection to gain a deeper understanding of current service provision within elite soccer developmental environments from the perspective of players, caregivers, practitioners, and support staff.

## Figures and Tables

**Table 1:** Participant demographics.

	Categories	Responses (*n*)
Sex	Male	232
	Female	129
Academy category	Category 1	75
	Category 2	75
	Category 3	210
Age group	FP	142
	YDP	218
“On average, how many hours does your child/dependent train per week? (include all fitness related activities, both at and away from the sporting club)”	<3 h	0
	3–4 h	2
	4–5 h	20
	5–6 h	28
	6–7 h	51
	7–8 h	58
	>8 h	201
“Does the sporting organization your child/dependent is with provide access to nutrition support for you and/or your child/dependent? Select all that apply”	Access to nutrition information relevant to healthy eating	154
	Access to nutrition information relevant to sports/training nutrition	152
	Access to group presentations by nutritionists/dieticians	88
	Individual consultations with nutritionists/dieticians	12
	Cooking classes	4
	Hand-outs	25
	Recipes	14
	None	126
“If not provided, what would you find beneficial for the sporting organization your child/dependent is with provide access to nutrition support for you and/or your child/dependent? Select all that apply”.	Access to nutrition information relevant to healthy eating	140
	Access to nutrition information relevant to sports/training nutrition	204
	Access to group presentations by nutritionists/dieticians	122
	Individual consultations with nutritionists/dieticians	155
	Cooking classes	46
	Hand-outs	85
	Recipes	136
	None	21

## Data Availability

All data underpinning this publication are openly available from the University of Northampton Research Explorer at doi:10.24339/9b98137d-70c7-4f96-a332-527bb99a4d46.
